# Illumina Sequencing Reveals the First Near-Complete Genome Sequence of *Ugandan Passiflora Virus*

**DOI:** 10.1128/MRA.00358-19

**Published:** 2019-05-16

**Authors:** E. K. Mbeyagala, S. Maina, M. W. Macharia, S. B. Mukasa, T. Holton

**Affiliations:** a National Semi-Arid Resources Research Institute (NARO-NaSARRI), Serere, Uganda; b Agriculture Victoria, Horsham, Victoria, Australia; c Biosciences eastern and central Africa-International Livestock Research Institute, Nairobi, Kenya; d School of Agricultural Sciences, Makerere University, Kampala, Uganda; KU Leuven

## Abstract

Here, we present the first near-complete genome of *Ugandan Passiflora virus* (UPV) sequenced from a symptomatic sample of KH7 passion fruit (Passiflora edulis Sims) variety. UPV had limited amino acid identity with other potyviruses known to cause passion fruit woodiness disease (PWD). The closest relationship (71.2% amino acid similarity) was with Bean common mosaic necrosis virus.

## Abstract (precis)

Here, we present the first near-complete genome of *Ugandan Passiflora virus* (UPV) sequenced from a symptomatic sample of KH7 passion fruit (Passiflora edulis Sims) variety. UPV had limited amino acid identity with other potyviruses known to cause passion fruit woodiness disease (PWD).

## ANNOUNCEMENT

Passion fruit woodiness disease (PWD) is globally the most economically important disease of passion fruit (Passiflora edulis Sims) (1). PWD is caused by several potyviruses (2–4). Recently, Ugandan Passiflora virus (UPV; genus Potyvirus, family Potyviridae) was reported to cause PWD in Uganda (5). To develop accurate viral diagnostic tools, full-genome sequence information is critical (6). However, there are no complete genome sequences of UPV currently in the GenBank database. A previous study in Uganda used partial sequencing to study coat protein and polyprotein genes of UPV (5). Here, we present the first near-complete genome sequence of UPV obtained from KH7 passion fruit variety collected from the Bushenyi district in western Uganda.

Five leaf samples were collected from a single plant belonging to KH7 variety and showing PWD-like symptoms. The KH7 plants were collected from a farmer’s field and maintained in an insect-proof screenhouse. Total RNA was extracted from the samples using a plant RNA miniprep kit (Zymo Research). An RNA quality check was done using Tris-acetate-EDTA (TAE)/formamide agarose gel electrophoresis (7). A single RNA sample with intact 18S and 28S subunits was selected for library preparation using a TruSeq stranded RNA sample preparation kit (Illumina). The final library concentration was verified using Qubit 2.0 (Invitrogen). Sequencing was done by MiSeq using a v3 kit (Illumina) with 2 × 301 cycles of paired-end (PE) reads.

The PE reads were subjected to CLC Genomics Workbench version 11.1 (CLCGW; CLC bio, Qiagen) for quality control by first trimming with previously described quality parameter settings (8). The trimmed PE reads were then subjected to *de novo* assembly using CLCGW with default settings. All contigs were subjected to the NCBI blast-2.2.29+ (9) search tool and then sorted by length. The open reading frame (ORF) for the contig of interest was predicted and annotated using Geneious (10).

The run yielded 8,432,604 raw reads, and 8,233,374 reads with a median length of 155 nucleotides (nt) remained after trimming. *De novo* assembly yielded 43 contigs of >1,000 bp. The contig of interest was 9,670 nt with 8,100,407 reads mapping to it with a coverage of 126,047×. The 9,670-nt contig (referred to here as KH7-1) had a G+C content of 40.73%. A BLASTN search revealed that KH7-1 resembled a *Ugandan Passiflora virus* isolate, UGM-19a (GenBank accession number FJ896000), with 99.36% nt identity over 1,718 bp. Also, KH7-1 had a single ORF comprised of 10 mature proteins typical of potyviruses (11, 12). Pairwise comparison of KH7-1 polyproteins using BioEdit Sequence Alignment Editor Version 7.0.4.1 (13) showed limited identity with other PWD-causing potyviruses. However, KH7-1 had 71.2% amino acid sequence identity with *Bean common mosaic necrosis virus* isolates (ANO46357 and ARI46489). Although the UPV sequences currently present in GenBank originate solely from Uganda, we propose a more neutral name for this virus (14), without geographical association, such as “*Passiflora virus*” (PV). This is because with increasing human movement and world trade involving plants/plant products, this virus might be found elsewhere (15). These findings have major implications for diagnosis and management of passion fruit viruses in Uganda and elsewhere.

### Data availability.

The sequence described here was deposited in GenBank under accession number MK110656. Raw data were deposited in the SRA under BioSample number SAMN10717829, which is part of BioProject number PRJNA513986.
